# Field Evaluation of a Coproantigen Detection Test for Fascioliasis Diagnosis and Surveillance in Human Hyperendemic Areas of Andean Countries

**DOI:** 10.1371/journal.pntd.0001812

**Published:** 2012-09-13

**Authors:** María Adela Valero, María Victoria Periago, Ignacio Pérez-Crespo, René Angles, Fidel Villegas, Carlos Aguirre, Wilma Strauss, José R. Espinoza, Patricia Herrera, Angelica Terashima, Hugo Tamayo, Dirk Engels, Albis Francesco Gabrielli, Santiago Mas-Coma

**Affiliations:** 1 Departamento de Parasitología, Facultad de Farmacia, Universidad de Valencia, Valencia, Spain; 2 Cátedra de Parasitología, Facultad de Medicina, Universidad Mayor de San Andrés, La Paz, Bolivia; 3 Salud Pública Veterinaria, OPS/OMS, La Paz, Bolivia; 4 Cátedra de Parasitología, Facultad de Farmacia y Bioquímica, Universidad Mayor de San Andrés, La Paz, Bolivia; 5 Unidad de Biotecnología Molecular, Facultad de Ciencias y Filosofia, Universidad Peruana Cayetano Heredia, Lima, Peru; 6 Bioquímica y Biología Molecular, Facultad de Ciencias y Filosofia, Universidad Peruana Cayetano Heredia, Lima, Peru; 7 Instituto de Medicina Tropical Alexander von Humboldt, Universidad Peruana Cayetano Heredia, Lima, Peru; 8 Organización Panamericana de la Salud, Lima, Peru; 9 Department of Control of Neglected Tropical Diseases, World Health Organization, Geneva, Switzerland; Swiss Tropical and Public Health Institute, Switzerland

## Abstract

**Background:**

Emergence of human fascioliasis prompted a worldwide control initiative including a pilot study in a few countries. Two hyperendemic areas were chosen: Huacullani, Northern Altiplano, Bolivia, representing the Altiplanic transmission pattern with high prevalences and intensities; Cajamarca valley, Peru, representing the valley pattern with high prevalences but low intensities. Coprological sample collection, transport and study procedures were analyzed to improve individual diagnosis and subsequent treatments and surveillance activities. Therefore, a coproantigen-detection technique (MM3-COPRO ELISA) was evaluated, using classical techniques for egg detection for comparison.

**Methodology and Findings:**

A total of 436 and 362 stool samples from schoolchildren of Huacullani and Cajamarca, respectively, were used. Positive samples from Huacullani were 24.77% using the MM3-COPRO technique, and 21.56% using Kato-Katz. Positive samples from Cajamarca were 11.05% using MM3-COPRO, and 5.24% using rapid sedimentation and Kato-Katz. In Huacullani, using Kato-Katz as gold standard, sensitivity and specificity were 94.68% and 98.48%, respectively, and using Kato-Katz and COPRO-ELISA test together, they were 95.68% and 100%. In Cajamarca, using rapid sedimentation and Kato-Katz together, results were 94.73% and 93.58%, and using rapid sedimentation, Kato-Katz and copro-ELISA together, they were 97.56% and 100%, respectively. There was no correlation between coproantigen detection by optical density (OD) and infection intensity by eggs per gram of feces (epg) in Cajamarca low burden cases (<400 epg), nor in Huacullani high burden cases (≥400 epg), although there was in Huacullani low burden cases (<400 epg). Six cases of egg emission appeared negative by MM3-COPRO, including one with a high egg count (1248 epg).

**Conclusions:**

The coproantigen-detection test allows for high sensitivity and specificity, fast large mass screening capacity, detection in the chronic phase, early detection of treatment failure or reinfection in post-treated subjects, and usefulness in surveillance programs. However, this technique falls short when evaluating the fluke burden on its own.

## Author Summary

A coproantigen-detection technique (MM3-COPRO ELISA) was evaluated in 436 and 362 schoolchildren of Huacullani, Bolivia, and Cajamarca valley, Peru, respectively. Classical techniques for egg detection were used for comparison. In Huacullani, using Kato-Katz as gold standard, sensitivity and specificity were 94.68% and 98.48%, respectively, and using Kato-Katz and COPRO-ELISA test together, they were 95.68% and 100%, respectively. In Cajamarca, using rapid sedimentation and Kato-Katz together, these results were 94.73% and 93.58%, respectively, and using rapid sedimentation, Kato-Katz and copro-ELISA together, results were 97.56% and 100%, respectively. There was no correlation between coproantigen detection by optical density (OD) and infection intensity by eggs per gram of feces (epg) in Cajamarca low burden cases (<400 epg), nor in Huacullani high burden cases (≥400 epg), although there was in Huacullani low burden cases (<400 epg). Six cases of egg emission appeared negative by MM3-COPRO, including one with a high egg count (1248 epg). The coproantigen-detection test allows for high sensitivity and specificity, fast large mass screening capacity, detection in the chronic phase, early detection of treatment failure or reinfection in post-treated subjects, and usefulness for surveillance programs. However, this technique falls short when evaluating the fluke burden on its own.

## Introduction

Fascioliasis is an important human and animal disease caused by the trematode species *Fasciola hepatica* and *F. gigantica*. At present, fascioliasis is emerging or re-emerging in numerous regions of Latin-America, Africa, Europe and Asia, both in humans and animals, a phenomenon which has partly been related to climate change [1]. Major human health problems are encountered in Andean countries (Bolivia, Peru, Chile and Ecuador), the Caribbean (Cuba), northern Africa (Egypt), western Europe (Portugal, France and Spain) and the Caspian area (Iran and neighbouring countries) [1]. Emergence, long-term pathogenicity [2]–[4] and immunological interactions [5], [6] prompted the WHO to include this disease among the so-called neglected tropical diseases (NTDs), which are chronic, debilitating, poverty-promoting and among the most common causes of illness in developing countries. Their control and elimination is now recognized as a priority to achieve the United Nations Millennium Development Goals and targets for sustainable poverty reduction [7], [8].

Among Andean countries, the highest human fascioliasis prevalences and intensities are encountered in the Northern Altiplano of both Bolivia [9], [10] and Peru [11], as well as in the Cajamarca valley (Peru) [12], where *F. hepatica* is the only fasciolid species present [13] and children and females are the subjects most affected [1]. Within the human fascioliasis high altitude transmission pattern related to *F. hepatica* transmitted by lymnaeid vectors of the *Galba/Fossaria* group in Andean countries, two different subpatterns have been distinguished according to physiographic and seasonal characteristics [1], [13]: a) the Altiplanic pattern, with endemicity distributed throughout an area of homogeneous altitude and transmission throughout the whole year caused by high evapotranspiration rates leading lymnaeid vectors to concentrate in permanent water bodies, e.g. the Northern Bolivian Altiplano [14]; b) the valley pattern, with endemicity distributed throughout an area of heterogeneous altitude and seasonal transmission related to climate, e.g. the Cajamarca valley in Peru [12], [15], [16].

In recent years, the availability of a very effective drug against fascioliasis, namely triclabendazole [17], prompted the WHO to launch a worldwide initiative against human fascioliasis [18], [19]. This initiative includes interventions in human fascioliasis endemic areas presenting different epidemiological situations and transmission patterns [1]. Bolivia and Peru are two of the countries selected for priority intervention due to the very large public health problem posed by this disease. Different pilot schemes were designed to assess the best control strategies according to the different epidemiological situations and transmission patterns. The Northern Bolivian Altiplano was chosen as an example of the Altiplanic pattern, while the Cajamarca valley was chosen as an example of the valley pattern.

An alternative to coprological egg detection is the use of immunodiagnostic tests based on the detection of anti-*Fasciola* antibodies and/or coproantigens released by the parasite. In the last decades, several ELISA methods based on the use of polyclonal and monoclonal antibodies have been reported to be useful for detection of *F. hepatica* and *F. gigantica* in the feces of sheep and cattle [20]–[23] and also rat feces [24]. Nevertheless, surveys on human fascioliasis have usually been made through various coprological techniques verifying the presence of eggs in stools [25] and antibody detection tests to confirm the diagnosis of human fascioliasis [26]. Among these techniques, classical coprological egg detection methods are the most frequently used [27]. However, so far, the use of coproantigen detection was applied to diagnose *F. hepatica* infection in patients in Cuba only [28], [29].

Enzyme-linked immunosorbent assay (ELISA) methods developed for determination of *Fasciola* coproantigens in stool samples from animals and humans provide an alternative to coprological examination [30], [31]. One of these methods is the MM3 capture ELISA (MM3-COPRO) test for fascioliasis diagnosis detection of fecal excretory-secretory antigens (ESAs) using a monoclonal antibody (mAb), whose usefulness for detection of *F. hepatica* and *F. gigantica* coproantigens in experimental and natural *Fasciola* infections of sheep and cattle has already been demonstrated [22], [32]. This test proved to be highly sensitive (confirmed by necropsy) and specific (no cross reaction was observed with antigens from other helminths), and allowed for the detection of *Fasciola* infections 1–5 weeks before patency in cattle. Furthermore, other researchers recently tested a commercial version of the test, and its appropriateness for the detection of *F. hepatica* infections in cattle was confirmed under field conditions [33]. The suitability of the MM3-COPRO method for detection of *Fasciola* coproantigens in both fresh and preserved stools from hospital patients has been demonstrated [34], but its applicability for detection of *F. hepatica* infections in humans under field conditions has not been proved.

An efficient coproantigen test for human fascioliasis diagnosis represents a valuable tool to facilitate population screening and post-treatment surveillance in control campaigns, above all in communities where people are reluctant to furnish blood samples due to ethnic/religious aspects. The aim of the present study is to evaluate the coproantigen technique MM3-COPRO ELISA under field conditions for human fascioliasis diagnosis in human hyperendemic areas of Andean countries, using classical coprological techniques for egg detection for comparison purposes (rapid sedimentation and Kato-Katz). Thus, two endemic areas were chosen: Huacullani (Bolivia) representing the Altiplanic pattern with high prevalences and intensities, and the rural areas of Cajamarca (Peru), representing the valley pattern with high prevalences but with low intensities. Results of the pilot intervention implemented in Huacullani to assess the feasibility of a strategy of large-scale administration of triclabendazole, with a focus on safety and efficacy, are included in another article [35].

## Materials and Methods

### Ethics statement

In Bolivia, the study was approved by the Comisión de Etica de la Investigación of the Comité Nacional de Bioética. In Peru, it was approved by the Comité Institucional de Etica of the Universidad Peruana Cayetano Heredia and the Comité de Ética of the Instituto Nacional de Salud.

All subjects involved provided written informed consent. Samples from children were obtained after consent from the children's parents, following the principles expressed in the Declaration of Helsinki. Consent was also obtained from the local authorities of the communities and heads and teachers of the school.

In Huacullani, activities were performed in collaboration with the Servicio Departamental de Salud La Paz and the Unidad de Epidemiología of the Bolivian Ministry of Health and Sports (MSyD). In Cajamarca, the study was done in cooperation with the Dirección Regional de Salud of Cajamarca, and the Estrategia Nacional de Zoonosis, Dirección General de Salud de las Personas, Ministerio de Salud (MINSA), Lima.

### Stool samples and coprological techniques

Coprological studies were carried out in the locality of Huacullani, which belongs to the municipality of Tiwanaku, third section of the province of Ingavi of the Departamento de La Paz, Bolivia. This locality is situated 85 km from the capital La Paz, at the western end of the so-called Tambillo-Aygachi corridor of the Northern Bolivian Altiplano. Huacullani has 2525 inhabitants, according to the last 2005 census of the Bolivian Instituto Nacional de Estadística. Stool collection was performed in the school of the locality and samples were obtained from a total of 436 children. Previous surveys in that locality showed very high prevalence rates of 38.2% in the year 1992, 31.2% in 1993, and 34.8% in 1996 in children, and 18.4% in 1996 and 11.8% in 1997 in total community surveys (children plus adults) [10].

Stool samples were also obtained in the Departmento de Cajamarca, Peru, which covers an area of around 35,400 km^2^ in the northern Andean part of Peru and is inhabited by 1,416,000 people. This Department comprises 13 provinces and the province of Cajamarca in turn includes 12 districts [12]. A total of 362 fecal samples were obtained from children of the schools of Escuela de Varones del Distrito (Jesus district), Santa Rosa de Chaquil (La Encañada district), and Andres Avelino Caceres (Baños del Inca district). Previous surveys showed very high prevalences in that endemic area, with a mean of 24.4% and the maximum prevalence of 47.7% in Santa Rosa de Chaquil, the hitherto highest local prevalence detected in Peru [12].

Classical coprological techniques for egg detection were used for qualitative (rapid sedimentation and Kato-Katz) and quantitative (Kato-Katz) diagnosis. The combined use of highly specific techniques has been reported as a means of compensating the low sensitivity of the Kato-Katz technique [36]. Thus, identification of true positive and true negative cases was carried out by using two criteria: i) finding of *F. hepatica* eggs in feces; ii) egg finding plus COPRO ELISA test results. Applying the Kato-Katz technique, eggs were detected in fresh stools after analysis of three Kato-Katz slides (Helm-Test, AK test, AK Industria e Comércio Ltda, Belo Horizonte, Brasil) per sample, depending on the concentration of *Fasciola* eggs following WHO recommendations, using a template delivering about 41.7 mg of feces [37]. The average egg output was calculated as eggs per grams of feces (epg). Parasitological analysis was done microscopically by a trained parasitologist. Intensity of infection, measured as eggs per gram (epg), was used as an indicator of *F. hepatica* burden in infected subjects. Kato-Katz was used in both study areas and rapid sedimentation was an additional test done in Cajamarca. In the case of the Huacullani samples, a single Kato-Katz slide was used for each sample. In the case of the Cajamarca samples, the rapid sedimentation procedure was applied and those fecal samples positive for the MM3-COPRO ELISA were also quantitatively analyzed by three Kato-Katz slides.

### Fecal sample procedures and analyses

Children were not included in the study if they presented any chronic or acute hepatic disease, pregnancy, breast-feeding, any acute infection within a week of enrolment, or receiving treatment for any other disease or condition. In Huacullani, at the time of the baseline survey (April 2008), the school population consisted of 459 children aged 5 to 14 years, who were all considered eligible for an interventional treatment study. A total of 447 children returned the plastic container. From these, 437 fecal samples from an equivalent number of children were examined (four children returned an empty plastic container, and six other children provided insufficient stool quantities to apply both Kato-Katz slide and COPRO ELISA). Thus, fecal samples were obtained from 437 children (220 males and 217 females) of 5–16 years of age (mean ± SD = 8.8±2.3). A clean, plastic, wide-mouthed, numbered container with a snap-on lid was given to every participant. All subjects were then asked to try to fill the container with their own feces and to return it immediately. One stool sample per subject was collected and personal data (name, sex, and age) were noted on delivery of the container. Samples were transported to the parasitological laboratory of the Faculty of Pharmacy, Universidad Mayor de San Andrés (UMSA), La Paz, within 1–3 h after collection. One aliquot was used to carry out the MM3-COPRO ELISA and another was preserved at 4°C to make the Kato-Katz slides. All Kato-Katz slides were made at the Laboratory of Parasitology of the Faculty of Medicine, UMSA, and were initially examined within 1 h of preparation to avoid overclarification of some helminth eggs.

In Cajamarca, at the time of the baseline survey (December 2007), the target population was 616 school children (age range 6–15 years old), with a coverage of 4.25% of the school children population and 0.86% of the overall population from the three aforementioned districts. Thus, in the present study, fecal samples were obtained from 362 children (264 males and 98 females), 7–15 years of age (mean ± S.D. = 9.9±2.2), by similar procedures. Samples were transported to Cajamarca city within 1–3 h after collection and stored at 4°C until being sent to the Laboratory of Parasitology at the Instituto de Medicina Tropical Alexander von Humboldt, Lima, where coproparasitological analyses were carried out. Both ELISA and Kato-Katz slides were applied to two aliquots of the material preserved at 4°C. A third aliquot was preserved in 10% formalin solution (1∶3) for subsequent egg detection by means of the rapid sedimentation technique [38].

To assure quality standards and possible handling differences, procedures in the two laboratories were implemented by the same personnel of the Valencia team in addition to the respective local personnel of each laboratory. In Cajamarca, stool samples were distributed into two groups according to the 400-epg threshold used for identifying high intensity infections [18]: a high burden group (≥400 epg) and a low burden group (<400 epg). However, in Huacullani, as precautionary measure, a lower threshold (300 epg) was requested to be applied by Bolivian health responsibles to distinguish between samples whose respective infected children were in need to be hospitalized for prevention follow up of potential post-treatment colics, and samples whose respective infected children were not hospitalized and were treated on an out-patient basis [35].

### Statistical analyses

Statistical analyses were done using PASW 17 software. For the evaluation of categorical variables, the chi-square test or Fisher's exact test was used. Bivariant correlations (Pearson's correlation) were calculated to assess the relationship between optical density (OD) and epg of *F. hepatica*. A P value below 0.05 was considered significant.

Theoretical positive predictive values (PPV) and negative predictive values (NPV) were calculated from sensitivity and specificity values obtained using only classical coprological tests for the identification of *F. hepatica* eggs in feces as “gold standard”. The following formulas were used for their calculation:
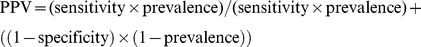


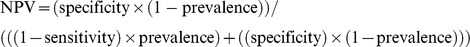



### MM3-COPRO ELISA detection of *Fasciola hepatica* coproantigens in fecal samples

The MM3-COPRO ELISA kits were prepared and tests performed as previously described [22], [32], [34]. Kits were provided by Dr. F.M. Ubeira (University of Santiago de Compostela, Spain). Briefly, polystyrene microtiter 1×8 F strip plates (Greiner Bio-One GmbH, Frickenhausen, Germany) were coated overnight with 100 µL/well of a solution containing 10 µg/mL of rabbit anti-*Fasciola* polyclonal IgG antibody in phosphate buffered saline (PBS) (wells from odd-numbered rows), or with 100 µL/well of a solution containing 10 µg/mL of IgG polyclonal antibodies from non-immunized rabbits (wells from even-numbered rows). Uncoated sites were blocked with 1.5% of sodium caseinate in PBS for 1 h at RT, and each fecal supernatant (100 µL) was then added in quadruplicate (2 odd-numbered wells plus 2 even-numbered wells), and incubated overnight at 4°C. After washing 6 times with PBS containing 0.2% Tween-20 (PBS-T), 100 µL of a solution containing 0.3 µg of biotinylated MM3 antibodies in PBS-T plus 1% bovine serum albumin (PBS-T-BSA) was added to each well and incubated for 1 hr at 37°C. After washing as above, bound MM3 antibody was detected by incubation, first with peroxidase-conjugated neutravidin (Pierce, Rockford, Illinois; dilution 1∶2000 in PBS-T-BSA) for 1 hr at 37°C, and then with the substrate (buffered H_2_O_2_ and o–phenylenediamine [OPD], Sigma-Aldrich, Madrid, Spain). After incubation for 20 min at RT, the reaction was stopped by addition of 3N H_2_SO_4_. Finally, OD was measured at 492 nm. The OD value for each sample was calculated as OD1–OD2, where OD1 is the mean for the 2 even-numbered wells (coated with anti-*Fasciola* polyclonal antibodies), and OD2 is the mean for the 2 odd-numbered wells (coated with irrelevant polyclonal antibodies). The OD value for each sample was calculated by subtracting the OD of the blank well from the OD of the test well using the cut-off point 0.097 [34].

## Results

Diagnostic parameters of the MM3-COPRO ELISA were estimated by (i) only choosing coprology as the “gold standard” assay to detect *F. hepatica* infection in humans, and also by (ii) considering results of both coprology and COPRO ELISA together. Positive cases of the MM3-COPRO ELISA and egg detection techniques of *F. hepatica* infection and performance characteristics of the MM3-COPRO ELISA according to study site are summarized in Table 1.

**Table 1:** Performance characteristics of MM3-COPRO ELISA by study site.

Endemic area	positive cases by MM3-COPRO	positive cases by egg detection	MM3-COPRO +	MM3-COPRO +	MM3-COPRO −	MM3-COPRO −	Sensitivity	Specificity	KATO-KATZ			
	%	%	Egg detection +	Egg detection −	Egg detection +	Egg detection −	%	%	N^a^	AM ± SD	Range	GM
Huacullani (N^b^ = 436)	24.77	21.56	89	19	5	324	94.68 (*)	98.48 (*)	94	334.98±92.56	24–8088	142.17
							95.68 (**)	100 (**)				
Cajamarca (N^b^ = 362)	11.05	5.24	18	22	1	321	94.73 (*)	93.58 (*)	17	116.47±84.80	16–376	89.80
							97.56 (**)	100 (**)				

Positive cases (%) of MM3-COPRO ELISA and egg detection techniques of *Fasciola hepatica* infection.

N^a^ = total number of positive children with *Fasciola* infection by Kato–Katz.

N^b^ = number of children analyzed.

AM = arithmetic mean; GM = geometric mean.

Identification of true positive and true negative cases was carried out by using two criteria:

i) finding of *F. hepatica* eggs in feces (*); ii) egg finding plus COPRO ELISA test results (**).

### Studies in Huacullani, Bolivia

Huacullani positive cases were globally 24.77% using MM3-COPRO ELISA and 21.56% applying an egg detection technique (Kato-Katz). No significant differences were encountered between either % (P = 0.093).

In this Bolivian locality, using Kato-Katz as gold standard, sensitivity and specificity were 94.68% and 98.48%, respectively, and using Kato-Katz and COPRO-ELISA test together as gold standard, sensitivity and specificity were 95.68% and 100%, respectively.

Of 436 samples assayed, 94 showed the presence of eggs through the Kato-Katz technique (21.56%). MM3-COPRO ELISA was positive in 108 samples (24.77%), which included samples with *Fasciola* eggs (89) and without *Fasciola* eggs (19), i.e. 82.40% of the children who were positive for the MM3-COPRO ELISA were also positive through the Kato-Katz procedure. It should be emphasized that there were five children shedding eggs with emissions of 48, 72, 96, 120 and 1248 epg, whose MM3-COPRO ELISA results were negative (1.14%). The stool sample showing 1248 epg was repeatedly re-analyzed and a negative result was always obtained with the MM3-COPRO ELISA test.

The geometric mean egg content in *F. hepatica* positive samples was 142.17 epg, and the arithmetic mean was 334.98 (with SD of ±92.56), with a range of 24 to 8088 epg (Table 1). In these samples from Huacullani, epg data were distributed into two groups: a high burden group (≥400 epg) and a low burden group (<400 epg) of samples.

**Figure 1:**
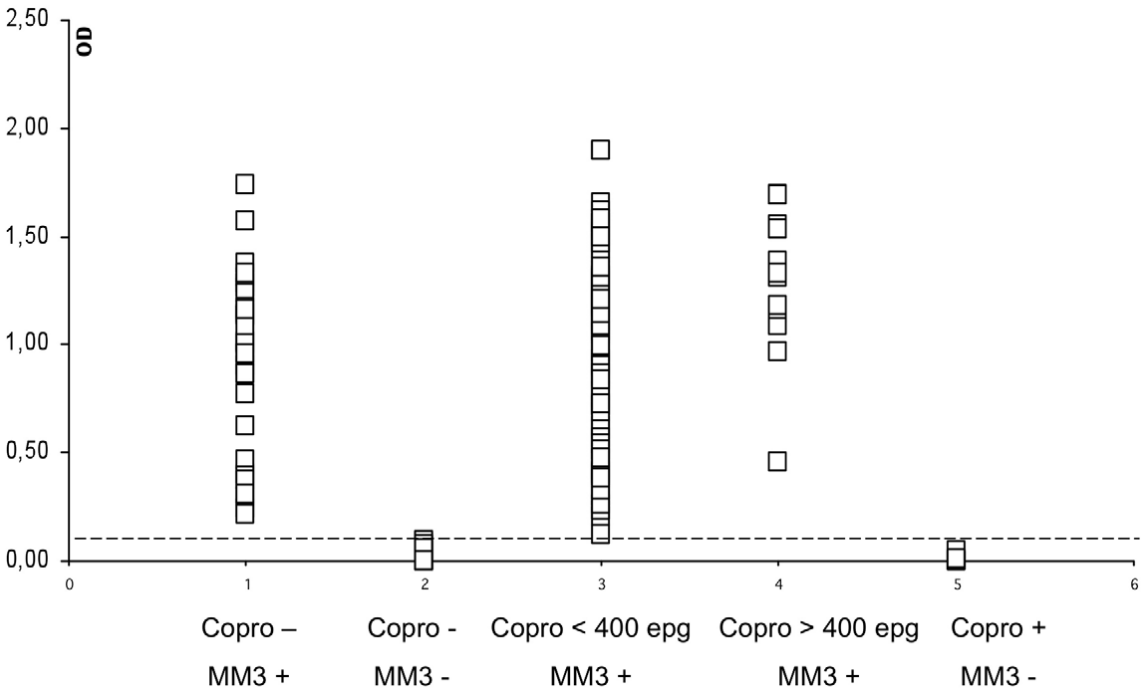
MM3-COPRO ELISA in feces from children (n = 436) from Huacullani (Northern Bolivian Altiplano). Data points represent the mean absorbance at 492 nm obtained from three replicates of each sample tested. The dotted line represents the cut-off value 0.097 units of OD at 492 nm.

The OD values obtained for individual *F. hepatica* positive and negative fecal samples from Huacullani are shown in Figure 1. Positive samples with *F. hepatica* eggs showed OD values above the cut-off value except in five cases (determined by the Kato-Katz technique).

**Figure 2:**
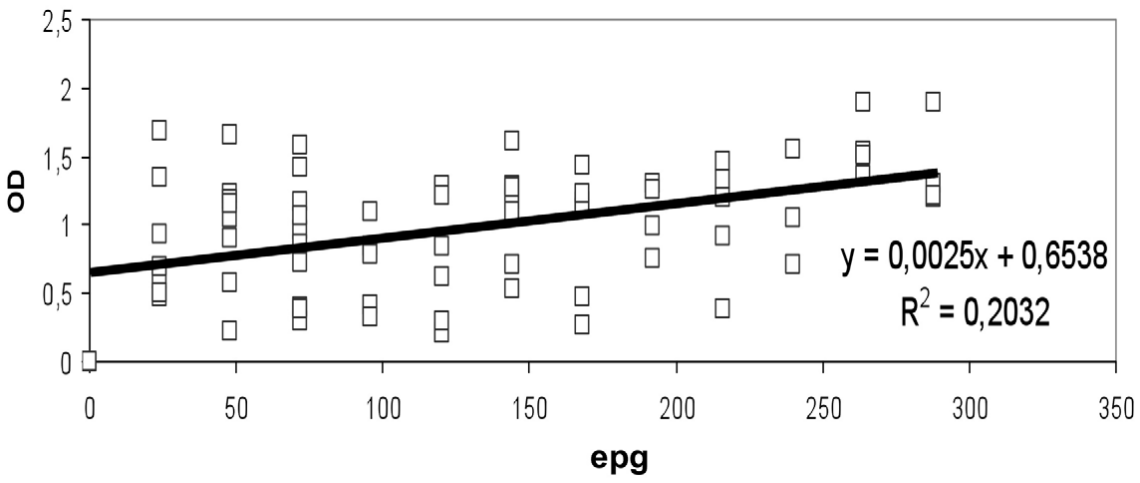
MM3-COPRO ELISA and intensity of *Fasciola hepatica* infection in the low burden group of Huacullani. Data points represent the mean absorbance at 492 nm from egg positive children from Huacullani. epg represents the egg count per gram of feces. The dotted line represents the cut-off value 0.097 units of OD at 492 nm.

**Figure 3:**
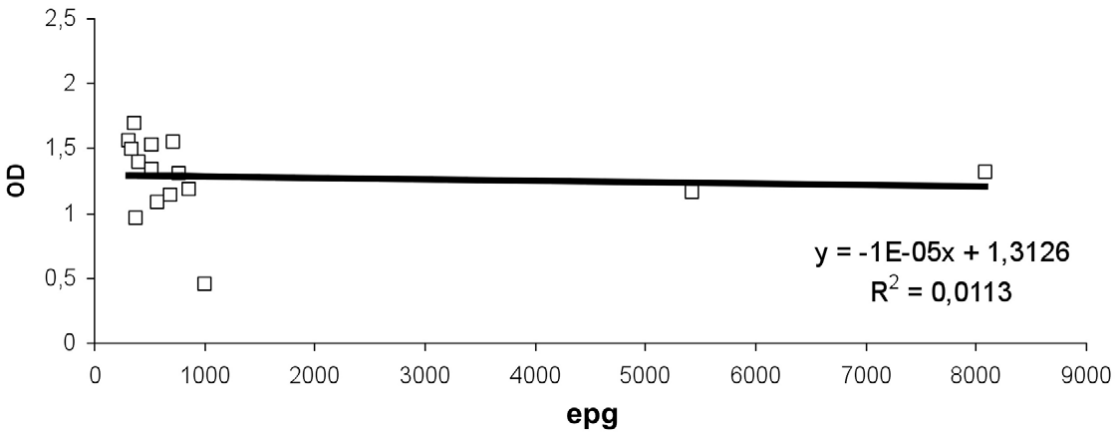
MM3-COPRO ELISA and intensity of *Fasciola hepatica* infection in the high burden group of Huacullani. Data points represent the mean absorbance at 492 nm from egg positive children from Huacullani. epg represents the egg count per gram of feces. The dotted line represents the cut-off value 0.097 units of OD at 492 nm.

In children who were positive in egg emission, the bivariant correlation between OD and epg data from low and high burden groups was carried out separately. A significant positive correlation was detected only between OD and low burden (*r^2^* = 0.20) (Figure 2), but no significant positive correlation was detected when considering OD and high burden (*r^2^* = 0.01) (Figure 3).

**Figure 4:**
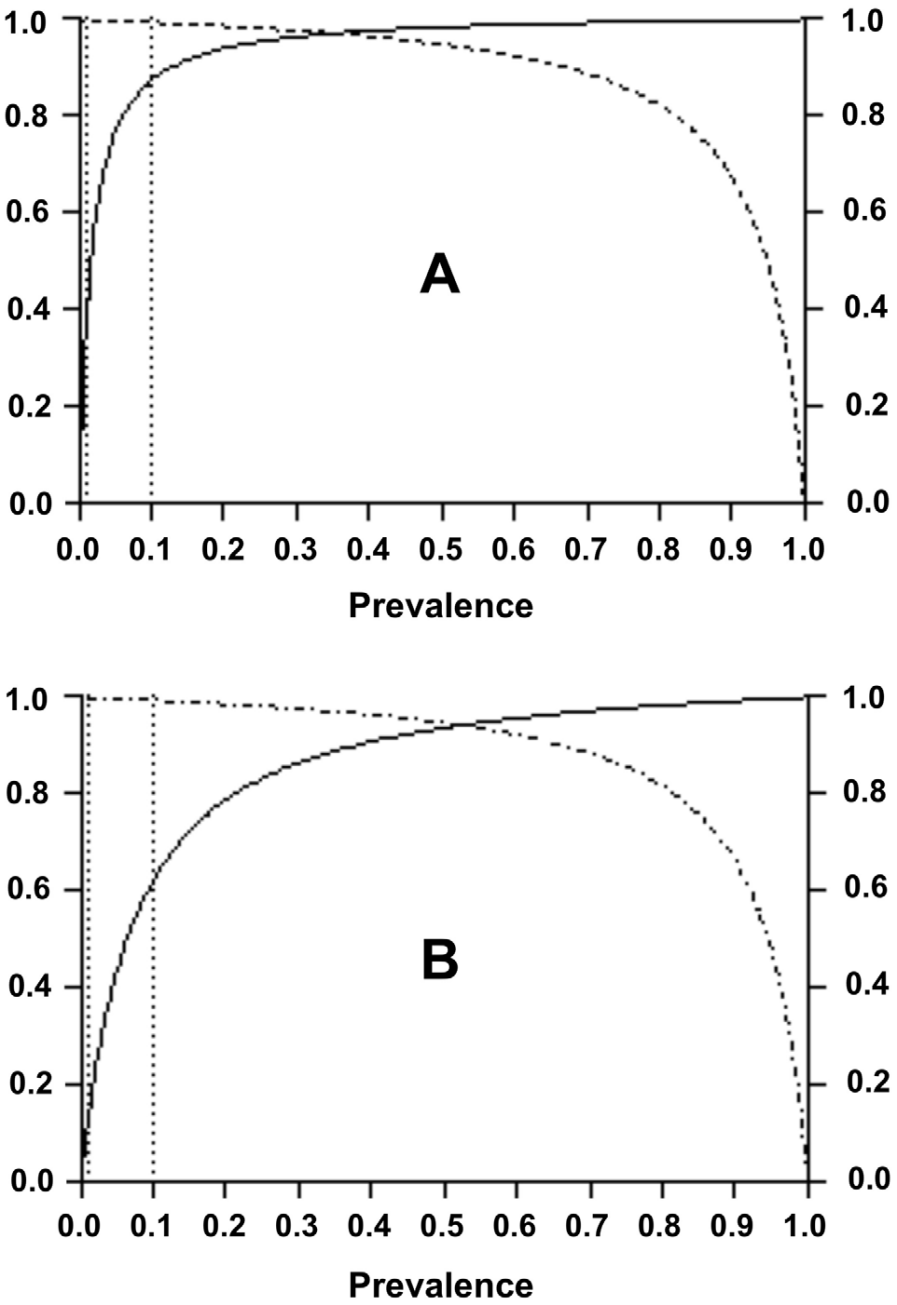
Theoretical PPVs and NPV values vs fascioliasis prevalence. Curves show the expected PPVs (continuous line) and NPV (dotted line) values in low (below 1%), medium (between 1% and 10%) or high (above 10%) prevalence scenarios (expressed in vertical lines) in Huacullani (A) and Cajamarca (B).

Theoretical PPVs and NPVs vs fascioliasis prevalence are represented in Figure 4A, showing the expected PPVs and NPVs depending on whether the test was used in low, medium or high prevalence scenarios in this Altiplanic highly endemic locality.

### Studies in Cajamarca, Peru

Cajamarca positive cases were globally 11.05% using MM3-COPRO ELISA and 5.60% employing egg detection techniques (rapid sedimentation and Kato-Katz). Significant differences were detected between both % (P = 0.007).

Differences between the two local patterns were detected, i.e. significant differences were found when comparing MM3-COPRO ELISA positive cases % from Huacullani and Cajamarca (P = 0.0025), and also when comparing egg detection positive cases % from Huacullani and Cajamarca (P = 0.001).

In Cajamarca, using rapid sedimentation and Kato-Katz together as gold standard, sensitivity and specificity were 94.73% and 93.58%, respectively, and using rapid sedimentation, Kato-Katz and copro-ELISA together as gold standard, results were 97.56% and 100%, respectively.

In this Peruvian locality, of 362 samples assayed, 19 showed the presence of eggs through the rapid sedimentation and Kato-Katz techniques (5.24%), whereas MM3-COPRO ELISA was positive in 40 samples, which included the samples with *Fasciola* eggs (18) and without *Fasciola* eggs (22), i.e. 45.0% of the children who were positive by MM3-COPRO ELISA were also positive through coprological egg detection procedures. Interestingly, one child shed eggs (by the rapid sedimentation technique) but was negative by MM3-COPRO ELISA. The remaining 321 MM3-COPRO ELISA negative samples, however, included 237 negative samples, and 84 positive samples for parasitic infections other than *Fasciola*. They involved one or more parasitic protozoans (*Blastocystis hominis, Chilomastix mensnilii, Giardia intestinalis, Entamoeba histolytica/E. dispar/E. moshkovskii, E. coli, Endolimax nana, Iodamoeba buetschlii*) and helminth species (*Strongyloides stercoralis, Ascaris lumbricoides, Trichuris trichiura, Enterobius vermicularis* and *Hymenolepis nana*).

The geometric mean egg content in *F. hepatica* positive samples from Cajamarca was 89.80 epg, and the arithmetic mean was 116.47 (with SD of ±84.80), with a range of 16 to 376 epg (Table 1), i.e. samples were all considered as belonging to the low burden group as their epg counts were <400.

**Figure 5:**
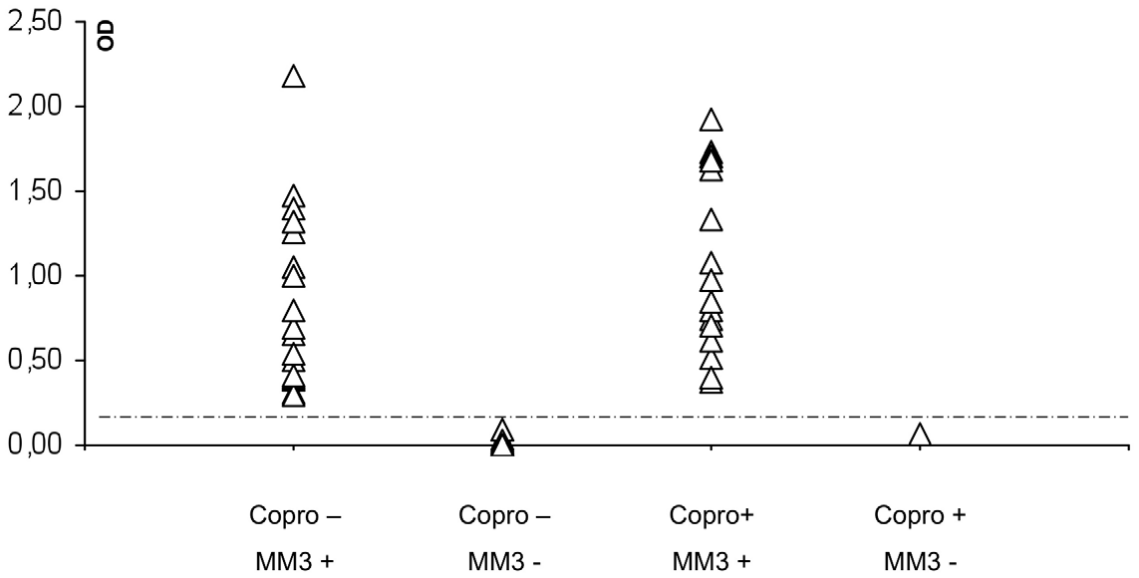
MM3-COPRO ELISA in feces from children (n = 362) from Cajamarca valley (Peru). Data points represent the mean absorbance at 492 nm obtained from three replicates of each sample tested. The dotted line represents the cut-off value 0.097 units of OD at 492 nm.

The OD values obtained for individual *F. hepatica* positive and negative fecal samples from Cajamarca are shown in Figure 5. Positive samples with *F. hepatica* eggs showed OD values above the cut-off value except in one case (determined by the rapid sedimentation technique).

**Figure 6:**
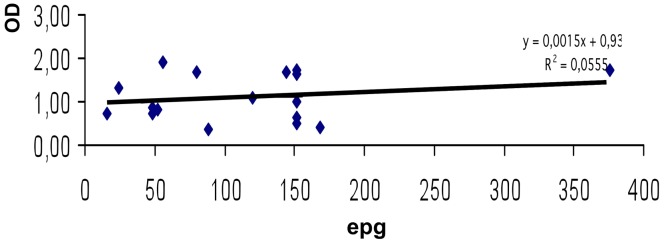
MM3-COPRO ELISA and intensity of *Fasciola hepatica* infection in the low burden group of Cajamarca. Data points represent the mean absorbance at 492 nm from egg positive children from Cajamarca. epg represents the egg count per gram of feces. The dotted line represents the cut-off value 0.097 units of OD at 492 nm.

In children who were positive in egg emission, the bivariant correlation between OD and epg data (low burden) was carried out. No significant positive correlation between OD and low burden (*r^2^* = 0.05) was detected (Figure 6).

Theoretical PPVs and NPVs vs fascioliasis prevalence are represented in Figure 4B, showing the expected PPVs and NPVs depending on whether the test was used in low, medium or high prevalence scenarios in this Peruvian highly endemic locality.

## Discussion

Sensitivity is defined as the proportion of people with the disease who have a positive test for the disease. A sensitive test will rarely miss people with the disease. Specificity is the proportion of people without the disease who have a negative test. A specific test will rarely misclassify people as having the disease when they do not [39]. Knowing true positive and true negative cases is essential when calculating sensitivity and specificity, respectively. The identification of true positive and true negative cases was carried out using classical coprological tests for the identification of *F. hepatica* eggs in feces. Nevertheless, in the case of human fascioliasis, the application of the rapid sedimentation or Kato-Katz techniques may result in false negative cases. The ethiological diagnosis based on egg detection in stools is complicated because parasite eggs are not found during the prepatent period [27], [40], when juvenile worms migrate through the intestinal wall to the peritoneal cavity (at one week), penetrate the liver parenchyma (at five to seven weeks), and pass into the biliary tract where they ultimately reach maturity (at two months or more). Previous studies have even estimated a period of at least three to four months to be necessary for *F. hepatica* flukes to attain sexual maturity in humans [27], [41]. Once the worms have matured, diagnosis still remains difficult because commonly employed microscopic techniques for quantitative diagnosis of *Fasciola* eggs are very specific but rather insensitive. In addition, in some cases diagnosis is also difficult during the biliary stage, due to the intermittent excretion of parasite eggs. Fecal egg counts are known to follow inter- and intraindividual variations in fascioliasis [42], [43]. In our case, we used the Kato-Katz technique as a “gold standard” because it is considered the best available for quantitative analysis, although taking into account that it is admittedly rather imperfect. Therefore, results from the rapid sedimentation were also considered to improve the first gold standard in Cajamarca, and the combination of results from both coprological methods and COPRO ELISA were used for both study areas.

When liver-flukes are located in the bile ducts, excretory-secretory (ES) products are released, being eliminated via feces. The detection of these products by means of a sandwich-ELISA reflects the installation of flukes in the bile ducts and the presence of the biliary stage of the disease [30]. No statistically-significant differences were detected between prevalence results obtained using egg detection techniques and the MM3-COPRO ELISA in Huacullani, where egg intensities are higher according to the typical feature of the Altiplanic pattern. On the contrary, such differences were detected in Cajamarca, probably as a consequence of the low egg intensities characteristic of the valley pattern, i.e. in Cajamarca low burdens are common and therefore the higher probability of infected subjects intermittently shedding very few eggs is higher, with the consequence that such cases go unnoticed.

Five and one cases of egg emission were negative using the MM3-COPRO ELISA in Huacullani and Cajamarca, respectively, including one case of very high egg count (1248 epg) in the Bolivian locality. Such cases pose a question mark. This result raises the question as to whether these false negative cases may be interpreted as being inherent to the kit design, or due to external factors not attributable to the ELISA kit. However, considering that there is broad experience in detecting *Fasciola* coproantigens in ovine and bovine samples, and that animals with egg emission were not found to be negative with the MM3 ELISA, it is unlikely that these false negative results were due to kit construction. Furthermore, a recent study has shown that the monoclonal antibody used in the MM3 assay recognizes L1 and L2 cathepsins [44], as does the ES78 in the FasciDig test [20], and there were no observations of false negative results with this test either. Three possible explanations include (i) an intermittent release of the ES products from the liver to the intestine through the bile ducts, (ii) spurious infections, and (iii) the existence of food remains in the intestine masking or interfering with the detection of the fluke ES antigens. The first does not seem to be the case, as previous studies using this and other kits do not indicate the emission of cathepsins L1 and L2 in either human or animal species to be intermittent (unlike with eggs). A spurious infection was excluded after meticuluous study of the aspect of eggs in each one of these children. A potential negative influence of high temperatures during the transport or/and an inadequate handling of the samples at a given moment throughout the whole procedure cannot be ruled out, although the relatively short storage period does not suggest a considerable denaturation of the L-cathepsins to have occurred.

In both Huacullani (representing the fascioliasis Altiplanic pattern) and Cajamarca (representing the fascioliasis valley pattern), cases of coproantigens present in the feces of humans without *F. hepatica* eggs in stools were detected. Previous time-course studies in animals on the detection of *F. hepatica* coproantigens by ELISA indicated that coproantigens were detectable prior to patency [45]. Furthermore, a marked increase in the levels of these coproantigens at the beginning of fecal egg output was observed [32]. Considering the positivity/negativity of MM3-COPRO ELISA and the presence/absence of eggs in feces, two situations were established in the current study: the Altiplanic pattern with a correlation between positivity of MM3-COPRO ELISA and the presence of eggs, and the valley pattern with a larger number of positive cases applying MM3-COPRO ELISA but without presence of eggs in feces.

The Altiplanic pattern, characterized by higher prevalences and intensities, showed no statistical differences between the percentage of children who were positive in coproantigens and eggs in feces. Thus, it may be concluded that the majority of children with liver-flukes in the biliary ducts shed eggs. Nevertheless, in the valley pattern, characterized by high prevalences but low intensities, differences were detected between the percentage of children who were positive to coproantigens and the reduced number of these children that shed eggs in feces. This suggests that children did not shed eggs or only a much reduced, undetectable number even despite presenting parasites in their biliary canals.

In Europe, for instance, the diagnosis of human fascioliasis is frequently established using serological tests, because the detection of *F. hepatica* eggs in stools is not always possible. Thus, in an epidemiological survey from 1970 to 1999 to record cases of human fascioliasis detected in the Limousin region (central France), egg detection in stools was positive in only 27.6% of a total of 711 persons with fascioliasis [46]. Future studies are needed in Cajamarca (and other endemic areas in valleys of the Andean countries) to verify whether in these cases the non-detection of eggs implies that the parasite (i) has not reached the biliary ducts or is located in the bile-ducts but oviposition has not yet started (suggesting a more or less recent infection) or (ii) oviposition is taking already place but with only very low egg numbers or with intermittent shedding (indicating that subjects present only one or a very few flukes in the chronic stage).

Negative results by the MM3-COPRO ELISA after treatment, which occurs approximately one to three weeks in animals, is usually accepted when determining the efficacy of anthelminthic treatment of biliary fascioliasis [22]. Contrarily, serological methods have limitations when determining the efficacy of anthelminthic treatment because the presence of antibodies indicates previous exposure to the parasite rather than the existence of a current infection. Additionally, after successful anthelminthic treatment, several months have to pass for serological antibody-detection tests to become negative. Hence, the detection of specific antigens in feces allows for the confirmation of a current infection, whereas antibody detection tests need to be complemented by another technique to confirm the results obtained in treated subjects. Future studies should be carried out to determine the time required for negativization of MM3-COPRO ELISA results after effective treatment in humans.

The MM3-COPRO ELISA is also a reliable method for detecting *F. gigantica* coproantigens in fecal samples from experimentally infected sheep [32]. Although most reported cases of human fascioliasis are caused by *F. hepatica*, infections by *F. gigantica* have also been reported [25]. The fact that the MM3-COPRO ELISA can detect infections by both species may be of great value to ensure diagnosis of human and animal fascioliasis in countries where *F. gigantica* predominates, or where both species of *Fasciola* are present [25], [34].

Determining a patient's parasitic burden is crucial given the necessity to monitor drug treatment in order to prevent a hepatic colic as the consequence of the massive expulsion of liver-flukes [18], similar to other helminth diseases [47]. The Kato-Katz is usually employed as a coprological quantitative technique. Nevertheless, this technique has a low sensitivity, and the elaboration of several slides from the same individual stool sample is indispensable. The application of the Kato-Katz technique in community surveys becomes problematic because (i) it is pronouncedly time consuming when the number of samples is high, (ii) microscopic egg count is also time consuming in cases of heavy egg burdens, and (iii) it requires an additional technique to increase the sensitivity in areas where subjects shed a very low number of eggs in an intermittent way.

Results obtained in the samples from Huacullani showed that the concentration of coproantigens in feces is correlated with epg in the low burden group (<300 epg). This result agrees with a previous study using the MM3-COPRO ELISA in cattle, which showed that the concentration of coproantigens in feces is also correlated with the number of flukes found in the livers of animals collected after slaughter [22], as well as with the results of positive correlation found with another coproantigen test in fascioliasis infected patients in Cuba [30]. Nevertheless, our findings in the high burden group (≥300 epg) showed that the concentration of coproantigens in feces is not correlated with epg. This result agrees with the absence of any correlation between egg shedding in human samples from Hospital patients, measured by the Kato-Katz technique, and coproantigen concentration, measured by the MM3-COPRO ELISA [34]. One possible explanation for this discrepancy may be that the positive cases analyzed in Cuba [30] probably corresponded to recent infections with less than a year of age (early chronic stage), whereas our samples were from patients with chronic infections, in which egg excretion is probably more erratic. It must be kept in mind that fasciolid flukes may survive for up to 13.5 years in humans, and the pattern of egg shedding is not linear but fluctuates between maximum and minimum values [43]. By comparison, the kinetics of coproantigen release versus the kinetics of egg shedding showed a similar pattern but with a two-week time lag in epg [32].

In Cajamarca, chronic fascioliasis in valley samples, coproantigen levels did not show a good correlation with epg. Therefore, the use of only one coproproantigen technique appears to be insufficient to evaluate the fluke burden.

In these hyperendemic areas, the number of subjects who participate in surveys of this kind is very large, which implies the problem of transporting and preserving the fecal samples, as the coproantigen degrades at ambient temperature within a few days and the fecal material cannot be treated with any classical fixative. The monoclonal antibody MM3 recognizes a single conformational epitope, located in *Fasciola* cathepsins L1 and L2, which are the main cysteine proteases produced by adult flukes gut [44]. The stability of the antigen was observed during a period of 5 weeks, except for samples preserved in CoproGuard, which were observed for 17 weeks. Comparison of the different preservation conditions revealed that even when maintained at 37°C, only the antigenicity of coproantigens in the samples diluted with CoproGuard did not vary throughout the observation period. In contrast, biocides such as sodium azide and thimerosal did not preserve the antigenicity, as the start signal decreased to approximately 30% by the end of the observation period. When the samples were maintained at 4°C, the *F. hepatica* coproantigens retained about 70% of their initial antigenicity after 5 weeks. However, the antigens are relatively stable in some stools. This suggests that degradation of MM3-recognized *Fasciola* coproantigens depends on the presence of particular protease species, or other factors, which differ for each patient [34].

Other studies have also referred to the stability of *Fasciola* coproantigens. The monoclonal antibody F10 recognizes a 26–28 kDa antigen which is a monomeric proteoglycan secreted and excreted from the tegument and the gut of the flukes. The antigenicity of that coproantigen was noted to be stable or even enhanced by the action of proteolytic enzymes found in the digestive tract and under a variety of standard laboratory storage conditions. Storage at various temperatures resulted in some break down of the protein. The storage of the purified protein at room temperature overnight gave rise to several new bands ranging from 8 kDa to 20 kDa. Incubation of the purified coproantigen at 4°C for two months resulted in a major band at 8 kDa and a minor band at 20 kDa which decreased in size with longer incubation. Storage of ES for more than three years resulted in a major band at 8 kDa not seen in fresh ES. All these bands were recognized by the monoclonal antibody. The 26–28 kDa band was always detectable and the smaller bands are lower in intensity, suggesting that the coproantigen is relatively stable during storage. Thus, that degradation probably represents a loss of carbohydrate, since antigenicity is maintained [48], [49].

One possible alternative would be freezing the samples at −20°C, but this poses the additional problems of (i) difficult and expensive transport of frozen samples to the laboratory where determination is to take place, and (ii) the non-appropriateness of frozen samples for the diagnosis of other parasite species present in coinfections. Another solution is the use of Coproguard, which has been demonstrated to be convenient for sample preservation in this kind of surveys including the application of a coproantigen-detection test [34].

In Huacullani and Cajamarca, the PPVs calculated for diverse epidemiological situations are very different. PPVs in hyperendemic situations were very high, making this test recommendable for such situations. Contrarily, NPVs calculated for diverse epidemiological situations are similar.

Current efforts for the control of human fascioliasis need diagnostic techniques which allow for high sensitivity and specificity, large mass screening, detection in the chronic phase, early detection of treatment failure or reinfection in post-treated subjects, and usefulness in surveillance programs. Our results indicate that a coproantigen-detection test such as MM3-COPRO ELISA fulfils all these aspects. It provides a good tool to detect biliary fascioliasis in humans under field conditions in Andean hyperendemic countries, including a higher sensitivity than egg detection techniques, especially in areas where burdens are usually low, such as in areas of the valley transmission pattern. Hence, the MM3-COPRO ELISA appears to be not only useful for individual diagnosis in hospitals, but also in human surveys in fascioliasis endemic areas characterized by low to high parasitic burdens.

The present MM3-COPRO ELISA validation is expected to facilitate the improvement of human fascioliasis diagnosis in endemic areas (a commercial version of the MM3-COPRO ELISA is today available). The practical application of this sensitive and convenient method for large scale surveillance in the control programs in the Northern Bolivian Altiplano and Cajamarca could improve screening of human fascioliasis in these endemic areas by detecting infected humans in the biliary stage of the disease, as a large number of samples can easily be processed. Keeping in mind that most affected subjects are usually children, the attainment of fecal samples is easier and faster than taking blood samples, which is considered invasive. The former does not pose difficulties for community elders, school head teachers and parents who usually give their consent. Moreover, to many of these indigenous communities, blood extraction is culturally not acceptable.

Furthermore, our experience in Huacullani and Cajamarca indicates that MM3-COPRO ELISA offers the easiest and fastest way to adequately face large mass screenings, by initially applying the coproantigen technique to all the coprological samples obtained in the community survey and thereafter applying the Kato-Katz technique only or first in coproantigen-positive samples. It is recommended to treat subjects with coproantigen-positive samples but with negative egg detection. This allows for a quick selected treatment action, lending the positive effects of (i) fast response in the communities surveyed that verify that infected subjects are treated within a few days after the survey, and (ii) reducing the probability of drug resistance appearance. The remaining coproantigen-negative samples may finally be analyzed for the eventual detection and subsequent selected treatment of very few subjects shedding eggs, although this last step will unavoidably be time-consuming.

Given the aforementioned advantages a coproantigen-detection test offers, one wonders why there are only relatively few of such tests for parasitic diseases affecting the digestive system available: amebiasis [50], giardiasis [51], opisthorchiasis [52], taeniasis [53], trichinelliasis [54], strongyloidiasis [55], hookworm infection [56]. Developing and/or improving highly specific coproantigen-detection test for diseases in which coprological diagnosis requires specialized personnel and time-consuming microscope work would evidently be welcome.
